# Fitness Effects of Chlorpyrifos in the Damselfly *Enallagma cyathigerum* Strongly Depend upon Temperature and Food Level and Can Bridge Metamorphosis

**DOI:** 10.1371/journal.pone.0068107

**Published:** 2013-06-26

**Authors:** Lizanne Janssens, Robby Stoks

**Affiliations:** Laboratory of Aquatic Ecology, Evolution and Conservation, University of Leuven, Leuven, Belgium; University of Sao Paulo, Brazil

## Abstract

Interactions between pollutants and suboptimal environmental conditions can have severe consequences for the toxicity of pollutants, yet are still poorly understood. To identify patterns across environmental conditions and across fitness-related variables we exposed *Enallagma cyathigerum* damselfly larvae to the pesticide chlorpyrifos at two food levels or at two temperatures and quantified four fitness-related variables (larval survival, development time, mass at emergence and adult cold resistance). Food level and temperature did not affect survival in the absence of the pesticide, yet the pesticide reduced survival only at the high temperature. Animals reacted to the pesticide by accelerating their development but only at the high food level and at the low temperature; at the low food level, however, pesticide exposure resulted in a slower development. Chlorpyrifos exposure resulted in smaller adults except in animals reared at the high food level. Animals reared at the low food level and at the low temperature had a higher cold resistance which was not affected by the pesticide. In summary our study highlight that combined effects of exposure to chlorpyrifos and the two environmental conditions (i) were mostly interactive and sometimes even reversed in comparison with the effect of the environmental condition in isolation, (ii) strongly differed depending on the fitness-related variable under study, (iii) were not always predictable based on the effect of the environmental condition in isolation, and (iv) bridged metamorphosis depending on which environmental condition was combined with the pesticide thereby potentially carrying over from aquatic to terrestrial ecosystems. These findings are relevant when extrapolating results of laboratory tests done under ideal environmental conditions to natural communities.

## Introduction

The widespread occurrence of interactions between stressors is an important threat to biodiversity [1]. This poses an enormous challenge for ecotoxicological research as the presence of interactions between pollutants and other stressors may strongly interfere with how pollutants tested in isolation may affect natural populations. One line of research focuses on interactions between pollutants themselves and developed models to predict mixture toxicity (reviewed in [2]), while another line of research focuses on interactions between pollutants and environmental conditions. Many studies have shown that the effects of pollutants may be magnified under suboptimal environmental conditions such as food shortage and suboptimal temperatures (reviewed in [3]–[5]). This is important to consider for ecological risk assessment, as in nature organisms often face suboptimal conditions while ecotoxicological studies typically expose test organisms under optimal environmental conditions [4]–[6].

Although interactions between pollutants and suboptimal environmental conditions can have severe consequences for the toxicity of pollutants and are widely documented (reviewed in [3]–[5]), their occurrence and fitness implications are still poorly understood and no specific predictive modeling framework for their combined impact has been developed [4]–[5], [7], partly because most empirical ecotoxicological studies considered only combinations with a single environmental variable and focused on one or two fitness-related variables. Therefore, it is hard to identify general patterns about the occurrence and impact of these interactions across environmental conditions and across fitness-related variables. With regard to the latter it is relevant to distinguish between fitness-related variables measured in the stage where the stressor is imposed and fitness-related variables measured in a later ontogenetic stage. Most animals have a complex life cycle with a larval and an adult stage that differ in morphology and habitat and that are separated by metamorphosis [8]. Yet, while several studies have looked at carry-over effects of larval exposure to a pollutant on adult traits (e.g. [9]–[12]), few studies have explored how combined exposure to pollutants and environmental conditions in the larval stage bridge metamorphosis and affect fitness-related variables in the adult stage. Some of them have documented interactive carry-over effects in the adult stage (e.g. [13]–[14]), while others did not (e.g. [15]–[16]).

Two important environmental conditions that often occur at suboptimal values are temperature [5] and food level [17]. Several studies documented interactions between suboptimal temperatures and pollutants (reviewed in [3]–[4]). Higher temperatures can make pesticides more toxic, for example through increased uptake rates that likely negate any increased detoxification capacity [6]. This is, however, not always the case as higher temperatures can result in a shortening of the aquatic stage, hence the duration of exposure to pollutants present in the water [18]. Also low food levels may increase the impact of pollutants as underfed animals have less resources available for physiological defense [19]. Even if an organism recovers from food shortage in the larval stage, there may still be carry-over effects on the adult's fitness [17].

In this study, we investigate the combined effect of exposure to the pesticide chlorpyrifos and suboptimal environmental conditions during the larval stage on a set of fitness-related variables measured in the larval stage (survival) and across metamorphosis (age and mass at emergence and adult cold resistance) in the damselfly *Enallagma cyathigerum*. Studies that consider interactions with pesticides across environmental conditions and across fitness-related variables, while crucial to identify general patterns about the occurrence and the impact of these interactions, are still rare, especially those that consider fitness effects of larval stress exposure in the adult stage. Given the aquatic larval stage and terrestrial adult stage of damselflies, such effects may couple aquatic and terrestrial ecosystems [20]. Besides age and mass at emergence, adult cold resistance is also an important fitness-related variable in damselflies since adult damselflies with a better cold resistance will better endure cold nights and likely be active earlier in the day. Therefore they can spend more time foraging and reproducing [20]. Surprisingly, as far as we know, no studies tested for effects of contaminants on cold resistance. We separately studied combined effects of chlorpyrifos and suboptimal temperatures on the one hand and combined effects of chlorpyrifos and low food levels on the other hand to explore consistent patterns across both environmental conditions and across the chosen set of four fitness-related variables. Chlorpyrifos is an organophosphate insecticide that is used in agriculture all over the world. Its mode of action is inhibiting the activity of acetylcholinesterase thereby disturbing signal transmission in the nervous system [21]. It is often found in ponds through runoff or direct application. Several studies have demonstrated negative effects of chlorpyrifos on non-target pond organisms, including aquatic insects (e.g. [22]) and fish (e.g. [23]).

## Materials and Methods

### Ethics statement

A collection and rearing permit for damselflies was obtained from ANB-Flanders.

### Collecting and housing

Twenty copulating females of the damselfly *Enallagma cyathigerum* (Coenagrionidae) were collected in “Het Stappersven”, a protected nature area without a known history of pesticide application in Kalmthout (Belgium). Females were transferred to the laboratory for egg laying and placed separately in vials with wet filter paper as oviposition substrate. Ten days after egg hatching, larvae were placed individually in 200 ml cups. All larvae were initially reared in a room with a constant temperature of 21°C and a photoperiod of L:D 14:10 hours. Damselfly larvae were fed ad libitum with *Artemia* nauplii five days a week (average daily dose = 1347, SE = 102, n = 15).

### Pesticide concentration

Based on a previous experiment, we chose a concentration of 1.0 µg/l chlorpyrifos, since this caused a growth reduction and only limited mortality in *E. cyathigerum* damselfly larvae (Lizanne Janssens, unpublished data). The chosen concentration is within the range of chlorpyrifos concentrations reported in nature [24]. We prepared the chlorpyrifos solution starting from a stock solution with a concentration of 10 µg/ml chlorpyrifos (kept in the dark at 4°C). This stock solution was a 100 times dilution of another stock solution containing 1 mg/ml chlorpyrifos dissolved in ethanol. The chlorpyrifos concentration of the stock solution at the start and at the end (3 months later) of the experiment was 1.000 mg/ml and 0.975 mg/ml, respectively. Samples were analysed by the independent research laboratory Lovap NV (Geel, Belgium) using gas chromatography in combination with mass spectrometry. The initial chlorpyrifos concentration in the experimental vials was 0.985 µg/l and after three days (just before renewal of the medium) the concentration was lowered to 0.472 µg/l, indicating that although the chlorpyrifos concentration fluctuated in time, the damselfly larvae were continuously exposed to the pesticide.

We used aerated dechlorinated tap water in the control treatment instead of a solvent control, since the amount of ethanol was only 1 µl/l exposure medium. A pilot experiment showed that there was no difference in survival, growth and development time of the study species at ethanol concentrations up to 2 µg/l (Lizanne Janssens, unpublished data). Moreover, the lowest NOEC reported for aquatic invertebrates is>10,000 times higher than the ethanol concentrations used in the pesticide treatment [25].

### Experimental setup

To test for the effects of pesticide exposure and food level on the one hand and pesticide exposure and temperature on the other hand as well as their potential interactions on life history traits (survival, development time, mass at emergence) and adult cold resistance, we set up two separate experiments. In the pesticide × food experiment, we crossed two pesticide treatments (control and 1 µg/l chlorpyrifos) and two food levels (low and high food) while in the pesticide × temperature experiment, we crossed the same two pesticide treatments (control and 1 µg/l chlorpyrifos) and two temperatures (18°C and 24°C). In both experiments, the exposure to the treatments started the day after the larvae molted into the final instar and lasted until emergence. The only exception was the temperature treatment which was introduced when larvae were 150 days old, ca. 50 days before their molt into the final instar. This was done to ensure larvae acclimated to their experimental temperature (18°C or 24°C) before the pesticide exposure started. In both experiments, the medium was renewed in all vials three times a week (static renewal experiment). The number of larvae tested at each treatment combination was 25 (total of 200 larvae).

In the pesticide × temperature experiment, larvae were transferred to incubators set at 18°C and at 24°C at day 150. These temperatures were chosen as they impose strong life history differences in coenagrionid damselfly larvae [26]–[27] and span the natural temperature regime in Belgium during the largest part of the larval growth season. The food level was the same as during the rearing period (*Artemia* nauplii ad libitum five days a week). For the pesticide × food experiment, larvae were kept at 21°C and fed *Artemia* nauplii six days a week at the high food level, and three days a week at the low food level. In addition, larvae at both food levels received five *Daphnia magna* juveniles three times a week.

### Response variables

**Figure 1:**
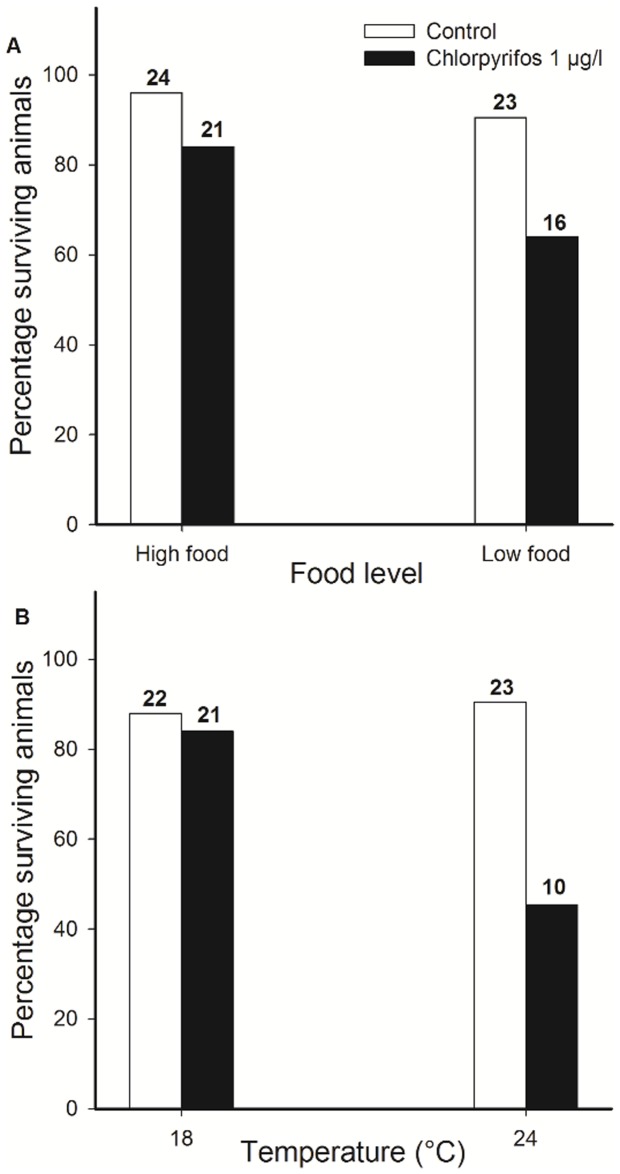
Effects of chlorpyrifos, food level and temperature on survival. Percentage of surviving *E. cyathigerum* damselflies as a function of exposure to the pesticide chlorpyrifos and food level (A) and temperature (B).

At the beginning of the exposure experiment, we weighed all animals to the nearest 0.01 mg in order to include this in the statistical models. We checked animals daily for survival and adult emergence. Development time was calculated as the number of days between molting into the final instar and adult emergence. One day after emergence, each adult was weighed to the nearest 0.01 mg and the sex was determined. Cold resistance was measured as chill coma recovery time, a widely used assay to estimate cold resistance in insects [e.g. 28]. We used a modified version of the protocol by Stoks and De Block [27]. We first placed adults individually in microcentrifuge tubes at 4°C for 2 h and then kept them on ice for 5 minutes. Afterwards each adult was placed on its back in a petri dish with roughened bottom at 21°C. We scored recovery times to the nearest second as the time taken for an animal to stand upright on its legs. Sample sizes slightly differed among treatment combinations because not all animals survived until emergence. None of the animals died during the cold resistance assay. The number of surviving animals is shown in Figure 1.

### Statistical analyses

We used a loglinear model to analyze the effects of the pesticide and the other treatment (food level or temperature) and their interaction on survival during the larval stage. To study the effects of pesticide and the other treatment (food level or temperature) and their interaction on development time, mass at emergence and chill coma recovery time, we performed separate ANCOVAs. When a test indicated a significant interaction, we performed Duncan's posthoc tests to further explore the interaction. For all tests, we included initial mass and sex in the statistical model; for chill coma recovery time, we also included mass at emergence. In the pesticide x temperature experiment there was no effect of the initial mass on the development time, so it was removed from the final model, resulting in an ANOVA. As sex is no focal variable of interest we will not show it in the figures. All tests were done in STATISTICA 11.

## Results

### Pesticide x food experiment

**Figure 2:**
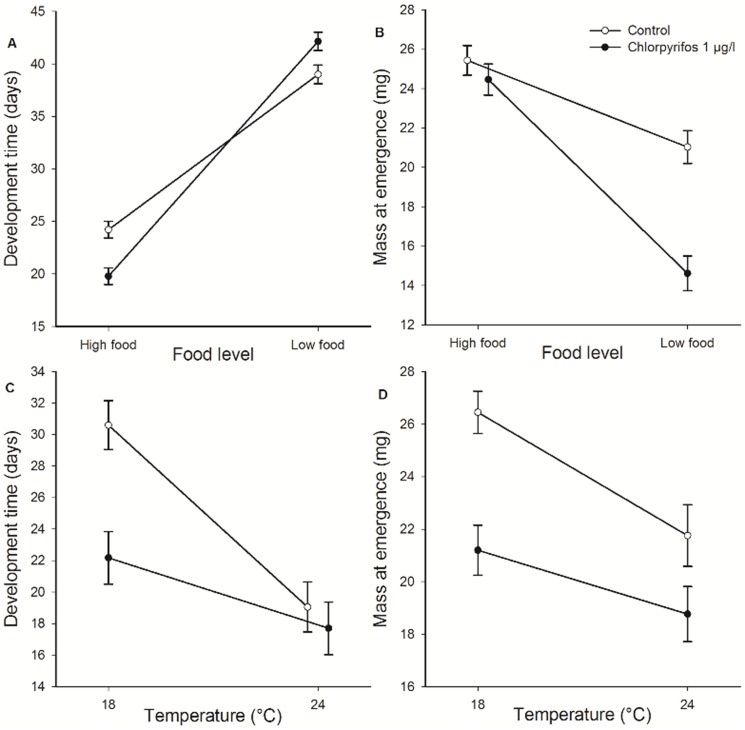
Effects of chlorpyrifos, food level and temperature on development time and mass at emergence. Mean larval development time (A, C) and mass at emergence (B, D) of *E. cyathigerum* damselflies as a function of exposure to the pesticide chlorpyrifos and food level (A, B) and temperature (C, D). Given are least-squares means ± 1 SE. Open symbols represent the pesticide-free control animals, closed symbols represent the pesticide-exposed larvae.

Survival was lower in the presence of the pesticide (χ^2^_1_ = 7.05, p = 0.0079) (Figure 1A). Although survival in the presence of chlorpyrifos seemed lower at the low food level (ca. 60% survival) than at the high food level (ca. 80% survival) there was no significant pesticide-by-food interaction (χ^2^_1_ = 0.13, p = 0.71). Larvae had much shorter development times at the high food level (F_1, 70_ = 411.87, p<0.001). There was a significant pesticide-by-food interaction for development time (F_1, 70_ = 16.61, p<0.001; Figure 2A): chlorpyrifos caused ca. 3 days longer development times at the low food level (Duncan: p = 0.029), yet ca. 5 days shorter development times at the high food level (p = 0.00051).

**Figure 3:**
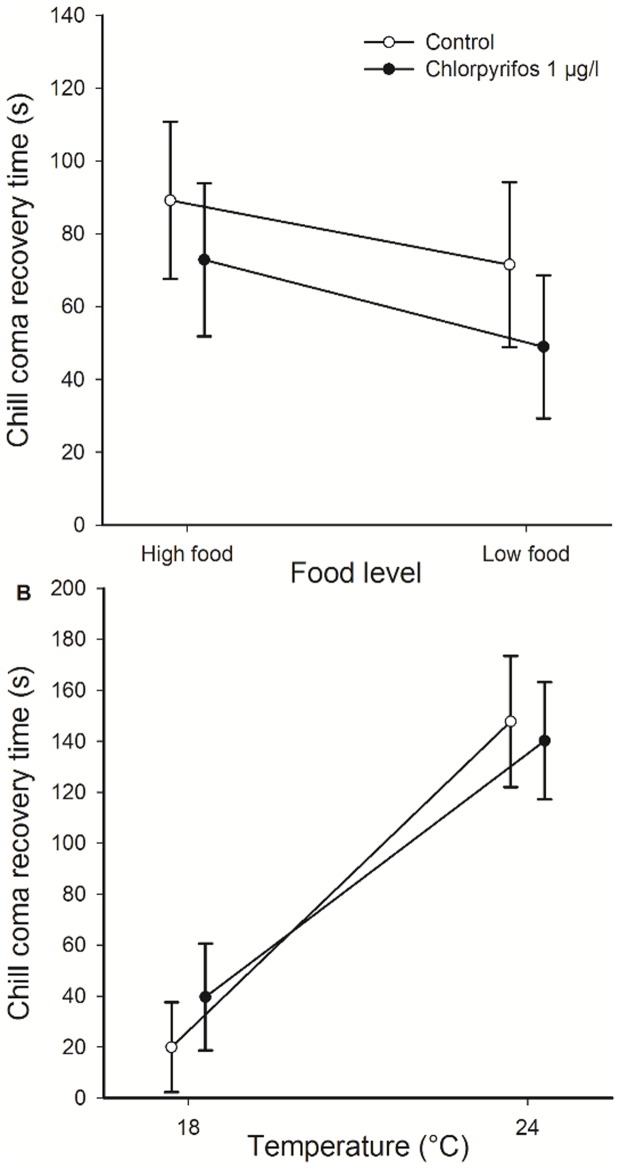
Effects of chlorpyrifos, food level and temperature on chill cold resistance. Mean chill coma recovery times of *E. cyathigerum* damselflyes as a function of exposure to the pesticide chlorpyrifos and (A) food level and (B) temperature. Given are least-squares means ± 1 SE. Open symbols represent the pesticide-free control animals, closed symbols represent the pesticide-exposed larvae.

Animals reared at the high food level were heavier at emergence (F_1, 71_ = 111.37, p<0.001). Chlorpyrifos exposure only resulted in lighter animals at the low food level (Duncan: p = 0.00012) and not at the high food level (p = 0.31) (Pesticide × food interaction, F_1, 71_ = 9.94, p = 0.0024; Figure 2B). The chill coma recovery time was shorter in adults reared as larvae at the low food level (F_1, 70_ = 5.84, p = 0.018). There was a trend for shorter recovery times in chlorpyrifos-exposed animals (F_1, 70_ = 2.87, p = 0.095). There was no interaction between pesticide exposure and food stress for cold resistance (F_1, 70_ = 1.08, p = 0.30) (Figure 3A). Heavier adults had shorter recovery times (F_1, 70_ = 7.22, p = 0.0090, slope ± 1 SE = −15.0±5.6).

### Pesticide x temperature experiment

Chlorpyrifos drastically reduced survival but only at 24°C (<50% survival) (Pesticide × temperature interaction, χ^2^_1_ = 4.01, p = 0.045) (Figure 1B). At 24°C, development times were shorter than at 18°C (F_1, 69_ = 24.49, p<0.001). Exposure to chlorpyrifos only resulted in a shorter development time at 18°C where the pesticide-exposed animals emerged ca. 9 days earlier (Duncan: p<0.001), and not at 24°C (p = 0.55) (Pesticide × temperature, F_1, 69_ = 4.78, p = 0.032; Figure 2C).

At 24°C animals emerged at a lower mass than at 18°C (F_1, 42_ = 10.0, p = 0.0029). Exposure to chlorpyrifos resulted in smaller animals at emergence (F_1, 42_ = 19.57, p<0.001). There was no interaction between pesticide exposure and temperature for mass at emergence (F_1, 42_ = 0.34, p = 0.56) (Figure 2D). The chill coma recovery time was only influenced by temperature (F_1, 43_ = 26.90, p<0.001) and not by the pesticide (F_1, 43_ = 0.076, p = 0.78) or their interaction (F_1, 43_ = 0.38, p = 0.54). Adults reared as larvae at 18°C had shorter recovery times than those reared at 24°C (Figure 3B).

## Discussion

### Survival

Neither food level nor temperature, had a significant effect on survival in the absence of chlorpyrifos, yet temperature modulated the pesticide effect on survival. Chlorpyrifos exposure (1 µg/l) throughout the final instar resulted in a reduced survival in the pesticide × food experiment (at 21°C) and in the 24°C treatment but not in the 18°C treatment in the pesticide × temperature experiment. The absence of a chlorpyrifos-induced mortality effect at the low temperature (18°C) has been observed before and can be explained by the reduction of an organism's metabolism at lower temperatures resulting in a decreased uptake (e.g. [29]). The higher cumulative mortality at 24°C illustrates that at higher temperatures the shortening of the exposure period may not always compensate for the increased toxicity of a pollutant, a mechanism that was recently shown for amphibians to alleviate the effects of a pollutant under global warming [18].

### Life history

Both development time ( = age at emergence) and mass at emergence, important fitness-related traits in damselflies [20], were affected by the manipulated environmental conditions (food level and temperature) and the pesticide and this often in an interactive way. Time until emergence was shorter at 24°C than at 18°C and longer at the low food level than at the high food level. This reflects the widespread patterns of a faster development at high temperatures and slower development at low food levels observed in many insects (reviewed in [30]), including damselflies (e.g. [13], [31]–[33]). Also the observed patterns in mass at emergence, being higher at the high food level and at lower rearing temperature support previous work in insects [reviewed in 24], including damselflies (e.g. [34]–[35]). The latter observation matches the temperature-size rule [36]: animals reared at a low temperature delay emergence for such a long time that they eventually emerge at a higher mass.

A striking finding was that the effect of pesticide exposure on development time strongly depended on food level and temperature. Animals reacted to chlorpyrifos by accelerating development but only at high food level or at the lower temperature. A faster development in response to pesticide exposure has been observed before in aquatic organisms (e.g. crabs [37] and tadpoles [14]). A possible explanation is that neurotoxic pesticides (such as chlorpyrifos) can excitate the central nervous system, resulting in a faster metamorphosis [37]. This response is likely adaptive as it shortens the larval stage, hence the duration of pesticide exposure [18]. When the pesticide-exposed animals were reared at the low food level, they instead lowered their development rate. This indicates that a life history acceleration in response to pesticide exposure is energetically costly. At the low food level, animals probably had a lower energy status and still needed to divert energy to costly defense and detoxification mechanisms which likely resulted in less energy available for development [19]. To our knowledge, no other studies exist that documented how food level modulates a pesticide-induced acceleration of development. Yet, in a study in tadpoles it was shown that carbaryl exposure only resulted in faster development in mesocosms with a low density of tadpoles, thus with a higher per capita food availability [38]. The absence of a pesticide-induced reduction of larval development time at the high temperature probably reflects a lower limit to the length of the final larval instar, which already was short in the control animals reared at the high temperature.

Chlorpyrifos exposure resulted in smaller adults except in animals reared at the high food level in the pesticide × food experiment. In aquatic organisms, it has been observed before that animals emerge at a smaller size due to pesticide exposure (e.g., damselflies [13], tadpoles [39], mayflies [40]). Such pesticide-induced mass reductions likely reflect a diversion of energy towards costly defense and detoxification mechanisms in combination with an increased metabolic rate (at high temperature) and the observed shortening of the larval stage (when larvae were exposed to the pesticide at low temperature). The observation that animals reared at the low temperature did strongly reduce larval development time while emerging at a smaller mass, indicates that animals prioritized to shorten the exposure period to the pesticide instead of investing in a higher mass at emergence. The absence of a pesticide-induced mass reduction at the high food level despite a pesticide-induced shortening of the final larval instar, may reflect the higher energy content of these larvae.

### Cold resistance

Both manipulated environmental conditions shaped cold resistance as measured by chill coma recovery times. This likely has fitness consequences because adult damselflies with a better cold resistance will better endure cold nights and likely be active earlier in the day. Consequently, they can spend more time foraging and reproducing [20]. Animals reared at the low food level had shorter chill coma recovery times, thus a higher cold resistance. The only other study that looked at the effect of larval food stress on adult chill coma recovery times found no effect (butterflies [41]). Although our results may seem contra-intuitive, other studies have shown that exposure to food stress may increase resistance to another stressor (e.g. in fruit flies [42]). The mechanisms causing such so-called cross-resistance are poorly understood. We hypothesize that stress proteins may play a role and that animals reared as larvae at the low food level had higher levels of the stress protein Hsp and therefore a better cold resistance in the adult stage. Indirect evidence for this hypothesis comes from the observation that food stress can indeed increase Hsp70 levels (e.g. birds [43] and fish [44]) and that adult damselflies with higher Hsp70 levels have a higher cold resistance [27]. Also mechanisms such as the reduction of metabolic rates under food stress (as observed in damselflies, [45]) may play a role to explain the cross-resistance [42].

Animals reared as larvae at the low temperature had shorter chill coma recovery times, hence a higher cold resistance. This is a general phenomenon observed in damselflies [27] and other insects (e.g. butterflies [41], crickets [46]) and reflects acclimation of the animals to the lower rearing temperature [41], [47]-[48]. Such acclimation effects have been explained by higher Hsp70 levels in animals reared at low temperatures [27].

The effect of larval exposure to chlorpyrifos on adult cold resistance was less pronounced and we could only detect a trend for a pesticide-induced increased cold resistance. Similarly, thermal stress during the larval stage was associated with a better resistance to malathion in adult mosquitoes [49]. This cross-resistance would again be consistent with an upregulation of Hsp70. Higher Hsp70 levels in response to chlorpyrifos exposure have indeed been shown in the study species [50], and in other taxa (e.g. fish [51] and flies [52]). This effect on cold resistance deserves further attention and goes against other studies on carry-over effects that show negative effects of larval pesticide exposure on adult fitness-related traits (e.g. [9]–[12]).

### Conclusions

In nature, organisms are often confronted with suboptimal environmental conditions in terms of food level [17] and temperature [5], and increasingly also with pollutants [53]. Moreover, these factors are known to frequently interact with each other (reviewed in [3]–[5]). Despite increasing attention, we still poorly understand the combined effects of environmental conditions and pollutants [5]. By studying combined effects of two environmental conditions and a widespread pesticide on a set of fitness-related variables, several important insights emerged.

First, our study showed that the effects of chlorpyrifos and the environmental conditions on three key fitness-related variables of damselflies (mortality, development time and mass at emergence) were rarely additive (but see the pesticide effect on mass at emergence in the pesticide × temperature experiment). Instead, most combined effects were interactive whereby the pesticide effects were strongly dependent (pesticide effects on mortality and development time only occurred at one temperature) and sometimes even reversed in comparison with the effect of the environmental condition in isolation (pesticide effects on development time as a function of food level) depending on the accompanying environmental conditions. Such reversal of pesticide effects under different levels of an environmental condition have been observed before. For example, carbaryl exposure resulted in slower tadpole development at low tadpole density and in faster development at high density [54]. Second, our study highlighted that different fitness-related variables responded differentially to the combination of an environmental factor and a pesticide. While food level did not change the adverse effect of chlorpyrifos on mortality, negative effects of chlorpyrifos on development time and mass at emergence were only present at the low food level. Furthermore, while temperature did not influence the negative effect of chlorpyrifos on mass at emergence, negative effects of chlorpyrifos on mortality were only present at the high temperature. Third, a given level of an environmental condition that is considered suboptimal given its isolated effect on fitness-related variables may not always have the same effect in the presence of the pesticide. A match occurred for low food levels: the low food level caused delayed emergence at lower mass in the absence of the pesticide, and this was also the case when the pesticide was present. Yet, while animals delayed emergence at 18°C in the absence of the pesticide, they emerged earlier in the presence of the pesticide. Fourth, while the interactive effect between the pesticide and the food treatment bridged metamorphosis as indicated by the interactive effect on adult mass, this was not the case for the interactive effect between the pesticide and the temperature treatment. Consequently, our study adds to the few other studies documenting interactive carry-over effects of pollutants and environmental conditions into the adult stage (see e.g. [13]–[14]), thereby likely generating effects across ecosystems [20]. Yet, our results also indicate that within the same study system the nature of the environmental condition may critically determine the existence of such interactive carry-over effects.

All four findings urge caution when making generalizations and interfere with the extrapolation of the results of laboratory tests done under ideal environmental conditions to natural communities. The daunting challenge in ecotoxicology will therefore be to construct a predictive framework for these interactions and to integrate these interactions in current risk assessment procedures [3]–[5], [7]. While modeling may prove rewarding in generating such predictive framework, more empirical studies that explore these interactions within and across life stages are needed before we could start identifying patterns using meta-analyses (e.g. [1]).
